# Reduced ovarian reserve among female offspring of consanguineous marriages in the Middle East—a mini review

**DOI:** 10.3389/frph.2025.1602090

**Published:** 2025-11-20

**Authors:** Rasha Bayoumi, Joy Riad, Sannidhi Pillai, Barbara Lawrenz, Human Fatemi

**Affiliations:** 1 School of Psychology, University of Birmingham Dubai, Dubai, United Arab Emirates; 2 ART Fertility Clinic, Abu Dhabi, United Arab Emirates

**Keywords:** ovarian reserve, infertility, parental consanguinity, fertility counselling, Middle East

## Abstract

**Background:**

Consanguineous marriages remain prevalent in many regions of the world, particularly within the Middle East, where reported prevalence exceeds 50% in countries such as Saudi Arabia, Oman, and the United Arab Emirates. Despite evidence that consanguineous marriages increase genetic risks through increased levels of homozygosity, which might lead to gene dysfunction, their impact on female fertility remains unclear. Although the data is limited and inconsistent, female offspring of consanguineous marriages appear to have a higher risk of reduced ovarian reserve compared to their peers from non-consanguineous marriages, with a more pronounced effect in young women.

**Aim:**

This mini review synthesizes current evidence on the relationship between parental consanguinity and ovarian reserve in female offspring to clarify existing findings and highlight research gaps.

**Methods:**

A systematic search of MEDLINE, Embase, and Web of Science was conducted up to March 2024 in accordance with the PRISMA guidelines. Studies evaluating ovarian reserve markers, including Anti-Müllerian Hormone (AMH) and Antral Follicle Count (AFC), in women with and without parental consanguinity were included. This review was registered with PROSPERO (Registration ID: CRD42022300162).

**Results:**

Three studies (*n* = 2,903) from Kuwait, the UAE, and Oman met the inclusion criteria. Two reported significantly lower AMH and AFC levels among women aged ≤35 years with parental consanguinity, whereas one found no significant association between parental consanguinity and ovarian reserve markers.

**Discussion:**

The current findings suggest that parental consanguinity may contribute to reduced ovarian reserve in female offspring; however, the data are not consistent. Differences in study design and degree of consanguinity may explain these inconsistencies. This review could be used to raise awareness about the potential influence of parental consanguinity on the reproductive health of their family's offspring, to encourage early counselling and proactive fertility assessment. The results present a call to action, highlighting the need for further research on this issue within the Middle East region, where consanguinity is highly prevalent.

**Systemic Review Registration:**

https://www.crd.york.ac.uk/PROSPERO/view/CRD42022300162, PROSPERO CRD42022300162.

## Introduction

Consanguineous marriages refer to the legal recognition of two people as spouses who are second-degree cousins or closer, and among Arabs, this includes double first cousins, first cousins, first cousins once removed, and second cousins (1, 2). Consanguineous marriages are common in countries where the extended family is the main social unit, and they developed from environmental and economic conditions prevalent in rural areas (3). Furthermore, they reflect the fundamental value system, as well as the identity of descent groups and the long-standing history of Arabian families. Contrary to prevailing beliefs in Western countries, consanguineous marriages are prevalent in many different parts of the world and are not limited to certain religious communities or rural areas, but are widely accepted in many populations (4, 5). Hence, they are more prevalent in the Middle East compared to other regions of the world (6). Interestingly, consanguineous marriages prevail in the Middle East despite urbanization and improvements in socioeconomic development, and have even increased in recent generations (3). Although, demographic studies indicate that these consanguineous unions are often linked to marginally smaller family sizes compared to non-consanguineous marriages (7). This pattern has been attributed to higher rates of reproductive loss, infant mortality, and subfertility observed among consanguineous couples (8, 9). Collectively, these findings emphasize that the reproductive impact of consanguinity extends beyond its well-established genetic risks.

Through their common ancestors, consanguine couples share genetic information: in case the spouses are first cousins, they share 1/8th of their genes and their children will be homozygous at 1/16th of all loci (10). The genetic risk for the offspring can be given by the inbreeding coefficient “F” (11) which denotes the fraction of loci at which the children of a consanguineous union are predicted to acquire identical gene copies from both parents. Consanguine spouses' offspring have a higher incidence and prevalence of autosomal recessive hereditary disorders, and, if multiple genes are affected, individuals may suffer from complex and overlapping symptoms (12, 13). Besides these often clearly obvious conditions, affecting the physical and mental development, it appears that female offspring of consanguineous marriages have a higher risk of reduced ovarian reserve compared to their peers from non-consanguine marriages with a pronounced effect in women below 35 years of age (14, 15); this condition, which might go unnoticed, could have a severe impact on the live and family planning of the affected individuals.

Infertility, caused by a reduced ovarian reserve, is usually considered physiologic in women of advanced age due to the age-related decline in the number and quality of the oocytes (16). However, reduced ovarian reserve causing infertility may be overlooked in young women due to the misconception that the ovarian reserve is only determined by age. Yet, current literature on this topic remains limited and fragmented, highlighting a significant gap in research that calls for further investigation. Therefore, the aim of this narrative review is to raise awareness about the implications of consanguineous marriages, more specifically with the increased risk of reduced ovarian reserve, in order to gain a better understanding of the multifactorial genetic disorder to minimize its impact on the female offspring and to take countermeasures to preserve fertility. With the goal of answering the overarching research question; How does parental consanguinity, compared with non-consanguinity, affect the ovarian reserve and fertility potential of women of a reproductive age in the Middle East?

## Materials and methods

The population of interest was women of a reproductive age with parental consanguinity, and the study populations consisted of clinical or community samples in any country. We included studies comparing women with “reduced ovarian reserve” and/or “ovarian deficit” to women with normal ovarian reserves. To reflect the wide range of outcomes in fertility research, we used multiple definitions for reduced ovarian reserves and consanguinity. The parameters used to define ovarian reserve were AMH and AFC, as these are the most accurate markers (17).

The exclusion criteria was as follows: (1) The study reported on non-human subjects only; (2) The study reported on male data only; (3) Consanguinity (CSG) was measured but there was no ovarian reserve outcome; (4) CSG and ovarian reserve were measured but the ovarian reserve outcome reported was not of interest; (5) Both CSG and ovarian reserve outcome measured but the association between them was not tested or reported; (6) No control group (not exposed to CSG); (7) CSG reported not of interest; (8) Only secondary data analysis; (9) Qualitative data only; (10) Related publication (same data set); and (11) Duplicate record (See Supplementary Appendix A). We did not impose any restrictions on language or publication date, and no restrictions on the types of study design eligible for inclusion.

## Search strategy

The search was conducted for articles that were published up until the 10th of March 2022, (updated 11th March 2024). The MeSH terms “reduced ovarian reserve” and “ovarian deficit” were used to identify studies examining the outcome. MeSH terms relating to “consanguinity” (CSG) such as “consanguine”, “cousin marriage” and “consanguineous marriage” were identified and combined with “OR”. Search terms for CSG were combined with search terms for ovarian using “AND”. The search strategy was replicated in Embase, MEDLINE, Web of Science, see Supplementary Appendix B for search strategy. Excel was used to import searches from each database, and after removing duplicates, studies were selected based on eligibility criteria. Disagreements at all stages were resolved through discussions among the reviewers.

## Data extraction and quality assessment

PRISMA Guidelines were adhered to while conducting the analysis and this manuscript was prospectively registered with PROSPERO (Registration ID: CRD42022300162). The extraction of data was conducted using a standard form. Information was extracted based on study design (cohort, case-controlled, cross-sectional), sample (location, size), the definition of risk factor, the primary outcome “reduced ovarian reserve”, confounders, data relevant to effect size calculation, and information required for quality assessment.

## Assessment of bias

We assessed study quality using the modified version of the Newcastle-Ottawa Scale (NOS) (18). Studies were classified as high, medium, or low quality. The assessment aimed to determine if the risk factor “consanguinity” was adequately evaluated, if controls were properly assessed when their selection was comparable to the exposed cases, and whether consanguinity was appropriately excluded in the control population. Two reviewers extracted data from each paper in duplicate. Each included paper underwent an independent NOS assessment by the two reviewers. Discrepancies were evaluated by a third reviewer and resolved through consultation with other members of the review group.

## Data synthesis

**Figure 1:**
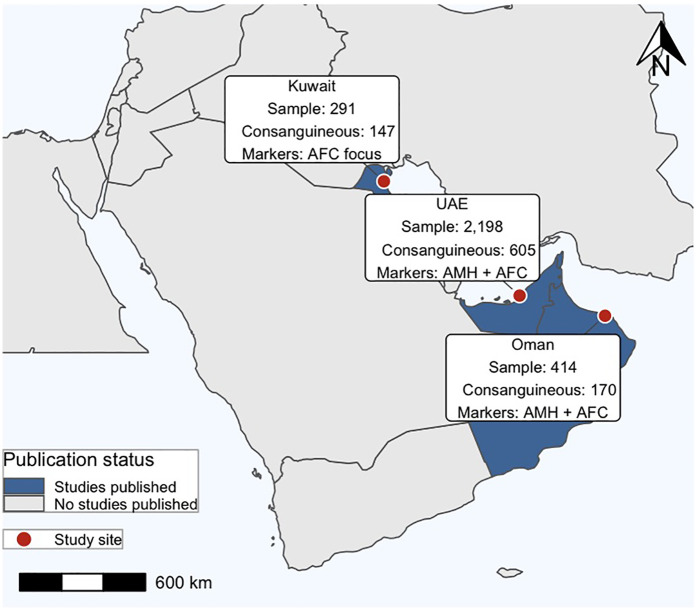
Geographic distribution of studies across the Arabian Peninsula. Study sites (red circles) are located in Kuwait, the United Arab Emirates (UAE), and Oman, with corresponding sample sizes, and markers analyzed (AMH and/or AFC).

A narrative review of the systematic evidence was conducted due to the insufficient number of primary studies to conduct a meta-analysis. The available evidence from the search and from known sources was summarized, and conclusions on the potential impact of the consanguinity on ovarian reserve was reported. A better understanding of the limited research in this area can be illustrated through a visual summary of the existing evidential data (See Figure 1).

## Results

### Search and identified studies

**Figure 2:**
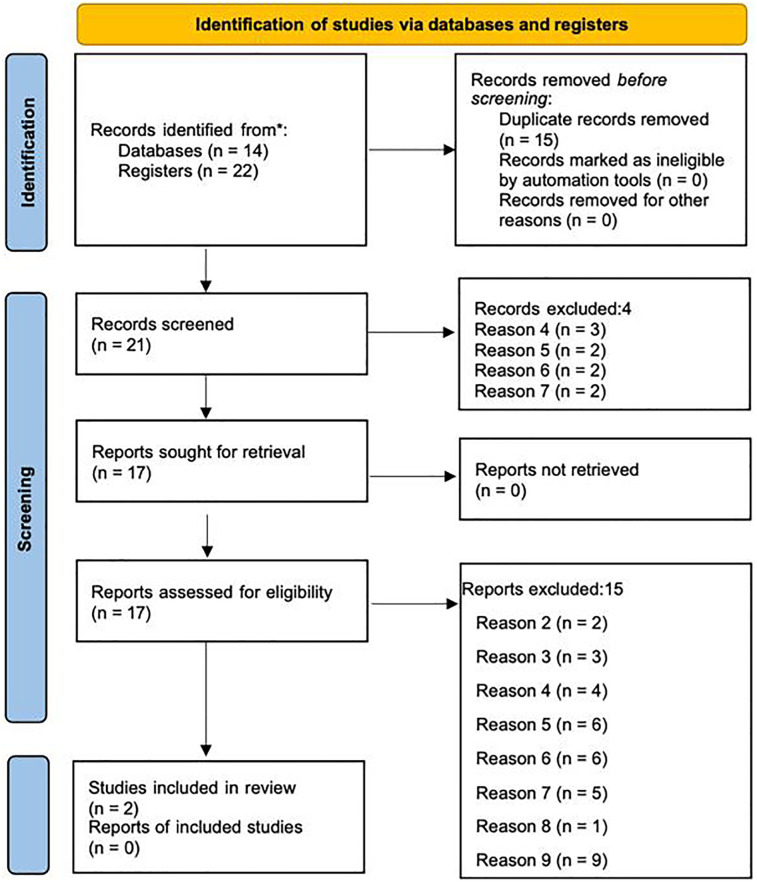
PRISMA 2020 diagram depicting the identification, screening, and inclusion process for studies assessing consanguinity and ovarian reserve markers (41).

We conducted a search on 10.03.2022 (updated on 11.03.2024) of Embase, Medline and Web of Science. This original search led to 36 results, of which 15 were removed as they were duplicates. We screened the remaining 21 articles. A search of the reference lists of the included studies and contact with authors resulted in no additional studies. Four studies were excluded at abstract, for the following exclusion criteria (see Supplementary Appendix A); (d) CSG and ovarian reserve were measured but the outcome of ovarian reserve was not of interest, (e) both CSG and ovarian reserved were measured but the association between them was not tested or reported, (f) no control group, and (g) CSG reported was not of interest, see Figure 2 PRIMSA diagram. Of the 17 full text articles assessed for inclusion, 2 primary studies met the inclusion criteria and were included in the narrative review, see Table 1. The 15 studies that were excluded at full text, were excluded for the following exclusion criteria; (b) male data only, (c) CSG was measured but there was on ovarian reserve outcome, (d), (e), (f), (g), (h) secondary data analysis only, and (i) qualitative data only, see Figure 2. The updated search revealed 3 more studies, 2 of which were excluded at abstract for exclusion criteria (d) and (e), and 1 that met the inclusion criteria and was included in this narrative review, resulting in a total of three studies included in this narrative review. All three studies were rated as moderate or high quality using the Newcastle Ottawa Scale. Due to the small number of studies, a meta-analysis was not possible, and we have therefore reviewed the studies narratively.

**Table 1:** Summary of included studies assessing the association between parental consanguinity and ovarian reserve markers in female offspring from Middle Eastern populations.

Author (year)	Country (sample)	Study design	Fertility-related parameters	Key findings	Direction of association	Comments
Seher et al. (14)	Kuwait (291)	Case study	AFC	Significantly lower AFC in women with consanguineous parents	↓ Ovarian Reserve	No association with BMI or cycle regularity
Melado et al. (15)	UAE (2,198)	Case study	AMH + AFC	Lower AMH and AFC in women ≤35 years with parental consanguinity	↓ Ovarian Reserve	Age-adjusted analysis confirmed significance
Al Saeghi et al. (19)	Oman (414)	Cohort study	AMH + AFC + FSH + LH + Estradiol	No significant difference in AMH/AFC between groups; partial AMH data due to funding	No Association	Possible underestimation/ misrepresentation due to missing data
Overall Summary	Kuwait, UAE, Oman (2,903)	Case & Cohort studies	AMH + AFC + FSH + LH + Estradiol	Two studies show reduced ovarian reserve linked to parental consanguinity; one shows no association	*Inconsistent*	Indicates heterogeneity and need for harmonized methodologies

AMH, anti-müllerian hormone; AFC, antral follicle count; FSH, follicle-stimulating hormone; LH, luteinizing hormone; BMI, body mass index.

### Narrative analysis of the included studies

Two case-controlled studies and one cohort study met the inclusion criteria and were included in the narrative review. These three studies encompassed a total patient sample of 2,903, of which 10% were in one study Seher et al. (14), 76% in another Melado et al. (15), and 14% in the final study Al Saeghi et al. (19). The studies involved women between the ages of 19 and 49 years Melado et al. (15), under the age of 40 years Seher et al. (14), or between 18 and 39 years old Al Saeghi et al. (19). In the first study Seher et al. (14), data was collected from Kuwait only. In the second study Melado et al. (15), data was collected in Abu Dhabi, United Arab Emirates, from patients native to several countries in the Arabian Peninsula (Yemen, Oman, Qatar, Bahrain, Kuwait, Saudi Arabia, and the UAE), who were undergoing fertility assessment/fertility treatment in Abu Dhabi. In the third study Al Saeghi et al. (19) data was collected from women in Oman only, who were undergoing treatment for infertility in various hospitals around the country.

The outcomes reported were Anti-Mullerian Hormone (AMH) and Antral Follicle Count (AFC) levels in the first study Melado et al. (15). In addition to AMH and AFC, the second study also reported outcomes such as menstrual irregularities, primary vs. secondary infertility, Follicle-stimulating hormone (FSH), luteinizing hormone (LH), and Estradiol concentration (11). The third study reported outcomes such as FSH, LH, Estradiol concentration, prolactin, thyroid stimulating hormone (TSH), AMH, and AFC Al Saeghi et al. (19). Measurements were taken for fertility assessment in all three studies. AFC was measured using a transvaginal scan while AMH concentrations were determined using the automated Elecsys immunoanalyser (Roche Diagnostics) (14, 20)/with an enzyme immunometric assay (19). Consanguinity was defined as parents being first-degree or second-degree cousins, and it was ascertained from self-report in the three studies.

All studies aimed to investigate the association between female parental consanguinity and reduced ovarian reserve among women from within the Arabian Peninsula. Results of the first study Seher et al. (14) indicated that consanguinity was strongly associated with a significant reduction of AFC count in this sample of Kuwaiti women. The median AFC of non-consanguineous daughters was 11, daughters from consanguineous parents had a median AFC of 7. Daughters of 1st degree cousins had a significantly reduced ovarian reserve (low AFC). Age, BMI, and regularity of menstrual cycle were not significantly associated with reduced ovarian reserve predictors.

Results of the second study Melado et al. (15), which consisted of a much larger sample (605 women from consanguine backgrounds and 1,593 from non-consanguine backgrounds), confirmed that female parental consanguinity is significantly associated with reduced ovarian reserve in the native sample of women from the Arabian Peninsula. Although median values for ovarian reserve markers appeared similar across both consanguine and non-consanguine groups, adjusted analysis by age demonstrated significantly lower AMH and AFC levels in women (≤35 years old) with parental consanguinity compared to those without such lineage (AMH: CV −0.10 ± 0.05, *P* = 0.035; AFC: CV −0.25 ± 0.08, *P* = 0.001). Notably, no significant differences were found in terms of body mass index, infertility duration, or smoking status between the two groups.

The results of the third study Saeghi et al. (19), focused on a cohort of 414 women from Oman, aged ≤39 years old (170 women with consanguineous parents and 244 women from non-consanguineous backgrounds). Assessment of ovarian reserve was carried out using AMH, FSH, and AFC (with the criteria for a low ovarian reserve defined as FSH levels ≥10 IU/L, AMH levels of <5 pmol/L, and an AFC of ≤7). Participants were classified into normal AFC (>7) and reduced AFC (≤7) categories. The findings revealed no statistically significant association between parental consanguinity and decreased ovarian reserve among the participants. Specifically, 15.0% of women with low AFC were from consanguineous parents, compared to 13% from non-consanguineous parents; a difference that did not reach statistical significance. Similarly, no significant disparities were observed in the prevalence of low AMH or elevated FSH levels between the two groups. However, it was only possible to estimate AMH levels in 40% of the women (181 of 414) due to the lack of funding. The study highlighted a significant relationship between the age difference that existed between women with low AFC and those with a normal AFC count. No statistical difference was found with women concerning low ovarian reserve and parental consanguineous marriage. The results also suggested no significant difference was observed between AFC levels and BMI.

## Discussion

Despite a worldwide occurrence of consanguineous marriages (21), our systematic literature review revealed only three studies which investigated the possible impact of parental consanguinity on the ovarian health of their female offspring. The studies included all together almost 3,000 women, recruited from fertility units in Kuwait (14), the United Arab Emirates (20) and Oman (19). Though all three studies included women from Middle Eastern countries, two publications (14, 20) described a reduced ovarian reserve in women from consanguineous parents; hence, the publication of Saeghi et al. (19) did not confirm these findings. Possible explanations for the discrepant findings are the use of different thresholds defining reduced ovarian reserve, the measurement of AMH only in part of the study population (19) and the inclusion of varying percentages of first- and second-degree parental consanguinity into the respective cohorts. A closer relationship (first-degree compared to second-degree) increases the pool of shared genes and sharing more genes implicitly increases the risk of undetected genetic abnormalities, some of which may have a deleterious impact on the ovarian reserve. Unfortunately, not all papers presented data on the distribution and their respective ovarian reserve of women with first- and second-degree parental consanguinity. For example, while Seher et al. (14) and Melado et al. (15) reported significant associations between parental consanguinity and reduced ovarian reserve, Saeghi et al. (19) did not, although this could be in relation to incomplete AMH measurements and underrepresented first-degree parental consanguinity. This heterogeneity across studies also resulted in complex comparisons, making it difficult to draw firm conclusions. Highlighting the need for more consistency in design rigor for future investigations.

The higher incidence of genetic diseases in offspring from consanguineous marriages due to elevated levels of homozygosity—which might lead to complete inactivation or dysfunction of genes—is widely recognized (7, 12, 13). However, the impact of consanguinity on the fertility of the offspring is still neglected. This is especially surprising given that societies in which consanguineous marriages prevail, are known for being pro-family, with strong family bonds and a desire for “big” families (8, 9). In Middle Eastern societies, childbearing is often considered essential for women (15), making the preservation of ovarian reserve a critical determinant of their fertility. Nevertheless, large-scale demographic surveys have reported that consanguineous couples tend to have fewer surviving children compared to non-consanguineous couples (8, 9). While this has traditionally been associated with higher rates of neonatal mortality and congenital disorders, it is also plausible that underlying reproductive factors, including reduced ovarian reserve in female offspring, contribute to this difference. These observations are consistent with the findings of our review, suggesting that the biological effects of consanguinity extend across generations, including both reproductive capacity and family size.

Aside from age and ethnicity (22–25), a variety of factors (e.g., environmental, iatrogenic, hormonal) can influence a woman's ovarian reserve. Recently, advanced genetic testing has revealed an increasing number of pathogenic mutations associated with premature ovarian insufficiency (POI) in women with and without parental consanguinity (26–29). With the current diagnostic tools, genetic factors seem to account for roughly 20 to 25% of POI cases (30). Whereas POI is an obvious condition as the affected woman's cycle will cease at a young age (<40 years), the presence of diminished ovarian reserve may go unnoticed, especially if the woman does not experience cycle irregularities, a history of/present infertility, or if she does not seek an assessment of her ovarian reserve. The “hidden” character of reduced ovarian reserve might also contribute to the—so far—limited identification of genetic factors involved in the pathophysiology of this condition.

It is important to acknowledge that parental consanguinity not only has a detrimental impact on female fertility, but also seems to cause male infertility (31–33). To shed further light on genetic factors that might influence the health of offspring of consanguineous marriages, some researchers support the notion of extending genetic studies in consanguineous populations to subjects without any clinical phenotypes (34). The need for this kind of study is supported by the fact that the data presented here are derived from women who attended fertility units, either because of an existing infertility or since they were interested in evaluating their ovarian reserve, which introduces the possibility of selection biases and limits generalizability. This raises concerns regarding external validity, as the findings may not be generalizable to women in the general population who do not attend fertility clinics (31). To obtain a more complete picture of the impact of parental consanguinity on male and female fertility, genetic testing should be expanded to a wider and potentially fertile population.

Another limitation pertains to measurement variability in both AMH and AFC levels that are subject to inherent sources of heterogeneity that may affect the accuracy and comparability of findings across studies (35). For AMH, values can differ substantially between the type of assay used, as variations in antibody specificity, calibration standards and analytical sensitivity can yield discrepant results from identical samples (36). Moreover, AMH concentrations are not entirely stable within individuals indicating intercycle fluctuations are influenced by age, reproductive stage and the menstrual phase (36–38). Similarly, AFC measurement is influenced by intercycle variability in follicle number and operator dependent factors such as equipment quality and examiner expertise (39). As a result, the variation within the included studies can limit the reliability and generalizability of the reported associations. Future research should aim to follow a “gold standard” of measures and rigorous methodological practices when investigating ovarian reserve to account for and limit the heterogeneity effects of existing empirical data.

However, until the effects of gene mutations and infertility are better understood, the narrative review presented here should raise awareness about the potential impact of parental consanguinity on offspring reproductive health and encourage individuals to actively seek counselling and assessment of their fertility status. Potentially, this review also urges the need for further investigation in understanding the scope of this topic globally. By systematically synthesizing disparate findings, this review provides a consolidated evidence base that highlights inconsistencies, identifies critical gaps, and sets a clearer research agenda than has previously been available, helping to better inform future research in this area. In case of a diagnosis of “reduced ovarian reserve”, existing fertility preservation techniques in form of oocyte and/or embryo cryopreservation after ovarian stimulation and/or ovarian tissue cryopreservation can be explored and performed, thereby forestalling the irredeemable loss of oocytes (39, 40).

## Data Availability

The original contributions presented in the study are included in the article/Supplementary Material, further inquiries can be directed to the corresponding author.
