# Supplementary material for: Reduced ovarian reserve among female offspring of consanguineous marriages in the Middle East—a mini review
Source: Front Reprod Health. 2025 Nov 20;7:1602090. doi: 10.3389/frph.2025.1602090 (PMC12675336; doi:10.3389/frph.2025.1602090)
Supplement: Supplementary file 1 [file Table1.docx]

**Appendix A: Exclusion criteria**

Reasons for exclusion of primary studies:

a. The study reported on non-human subjects only

b. The study reported on male data only

c. Consanguinity (CSG) was measured but there was no ovarian reserve outcome

d. CSG and ovarian reserve were measured but the ovarian reserve outcome reported was not of interest

e. Both CSG and ovarian reserve outcome measured but the association between them was not tested or reported

f. No control group (not exposed to CSG)

g. CSG reported not of interest

h. Only secondary data analysis

i. Qualitative data only

j. Related publication (same data set)

k. Duplicate record
