# Supplementary material for: Reduced ovarian reserve among female offspring of consanguineous marriages in the Middle East—a mini review
Source: Front Reprod Health. 2025 Nov 20;7:1602090. doi: 10.3389/frph.2025.1602090 (PMC12675336; doi:10.3389/frph.2025.1602090)
Supplement: Supplementary file 2 [file Table2.docx]

**Appendix B: Search Strategy**

The following databases were searched:

- Embase
- Medline
- Web of Science

The search strategy was to combine searches of:

- ‘Ovarian reserve’ and ‘Consanguinity’ free text terms and MeSH terms

**Ovarian Reserve Terms**

1. Ovarian reserve
2. Ovarian deficit
3. Reduced ovarian reserve

**Consanguinity Terms**

1. Consanguinity
2. Consanguine
3. Consanguineous
4. Consanguineous marriage
5. Cousin marriage
